# Tuberculosis recurrence in a high incidence setting for HIV and tuberculosis in Brazil

**DOI:** 10.1186/s12879-014-0548-6

**Published:** 2014-10-24

**Authors:** Gisela Unis, Andrezza Wolowski Ribeiro, Leonardo Souza Esteves, Fernanda Sá Spies, Pedro Dornelles Picon, Elis Regina Dalla Costa, Maria Lucia Rosa Rossetti

**Affiliations:** Hospital Sanatório Partenon - HSP, 3722, Bento Gonçalves Av, Porto Alegre, 90650-001 RS Brazil; Fundação Estadual de Produção e Pesquisa em Saúde - FEPPS/SES/RS. 5400, Ipiranga Av, Porto Alegre, 90610-000 RS Brazil; Universidade Federal do Rio Grande do Sul, CBIOT, Bento Gonçalves Av, Porto Alegre, 90000-000 RS Brazil; Programa de Pós-graduação em Genética e Toxicologia Aplicada-Universidade Luterana do Brasil - ULBRA, 8001 Farroupilha Av, Canoas, 92425-900 RS Brazil

**Keywords:** Tuberculosis, Recurrence, HIV, MIRU

## Abstract

**Background:**

To compare epidemiological data between recurrent cases after cure (RC), distinguishing relapse from reinfection, after dropout (RD) and new cases (NC) in an ambulatory setting in a TB-endemic country.

**Methods:**

Records of patients who started treatment for pulmonary TB between 2004 and 2010 in a TB clinic were reviewed. Epidemiological data were analyzed. Spoligotyping and MIRU patterns were used to determine relapse or reinfection in 13 RC available.

**Results:**

Of the eligible group (1449), 1060 were NC (73.2%), among the recurrent cases, 203 (14%) were RC and 186 (12.8%) were RD. Of RC, 171 (84.2%) occurred later than 6 months after a previous episode, 13 had available DNA, in 4 (30.7%) the disease was attributed to reinfection and in 9 (69.3%), to relapse. Comparing RC to NC, HIV (p < 0.0001) was independent risk factor for RC. When RC and RD were compared, alcohol abuse (p = 0.001) and treatment noncompliance (p = 0.006) were more frequent in RD.

**Conclusions:**

HIV is the sole more important associated factor for RC. This finding points the need to improve the approach to manage TB in order to decrease the chance for exposure especially in vulnerable people with increased risk of developing disease and to improve DOTS strategy to deal with factors associated to treatment noncompliance.

**Electronic supplementary material:**

The online version of this article (doi:10.1186/s12879-014-0548-6) contains supplementary material, which is available to authorized users.

## Background

Although many studies have been done about recurrence of tuberculosis (TB) following completion of treatment it is still a huge problem for public health in high burden countries, where no special attention is being given to this subject [1]. Treatments of recurrent episodes are often associated with drug resistance and low cure rates. The incidence and risk factors for recurrence, as well as the contribution of relapse or reinfection is being searched by several studies with different results [2]–[9]. Recurrence rates depend largely on TB incidence and HIV prevalence [3],[4]. In high incidence areas for TB, people who have been treated successfully are at higher risk of developing TB from reinfection than the general population and HIV are at more risk of reinfection than non HIV [9]–[11].

In 2010, the Brazilian Ministry of Health made changes in TB treatment, adding a fourth drug to the basic regimen because of increasing resistance and imposed direct observed treatment for multidrug resistant tuberculosis (MDRTB) as a rule to provide the drugs to ensure adhesion to treatment as an effort to decrease new cases and recurrence [12].

This study aimed to investigate recurrence after cure (RC), to distinguish relapse from reinfection and compare to new cases (NC) and recurrence after dropout (RD) in an ambulatory setting in a high burden area for TB and HIV in Brazil, describing epidemiological characteristics of these groups in order to bring new strategies to control recurrence, meaning primary and secondary preventive therapy, implementation of infection control measures in clinical and community settings and special care to vulnerable groups.

## Methods

### Study setting

The study was conducted in a reference Center for TB, placed at Hospital Sanatório Partenon in Porto Alegre city, Rio Grande do Sul, Brazil. It covers the east side of the city, counting about 180.000 inhabitants (12% of Porto Alegre population). The incidence of cases was on average 104.6 per 100.000 population per year (2004-2010). In the study area, the prevalence of HIV infection among new patients was approximately 23% [13].

### Ethics compliance

This study was approved by the ethical committee of Fundação Estadual de Produção e Pesquisa em Saúde - FEPPS/SES/RS (protocol number 02/2010).

### Study population and follow-up

All adult cases starting treatment for pulmonary tuberculosis confirmed by baciloscopy and/or culture between 2004 and 2010 were eligible for this retrospective analysis. New cases (NC), recurrence after cure (RC) or recurrence after dropout (RD) were analyzed for HIV status, and other epidemiological factors such as: sex, age, alcohol abuse, and drugs, smoking and treatment compliance. If recurrence after cure, period between the two episodes and cultures done in the first and second episode were also analyzed. All retreatment patients after cure with a positive culture in the first and second episodes available were identified for relapse or reinfection by molecular techniques.

### Definitions

A TB case was defined as pulmonary tuberculosis confirmed by baciloscopy and/or culture with radiological and clinical findings suggestive of TB. Recurrence of TB was defined as a second episode of TB occurring after a first episode had been considered cured.

Disease attributable to reinfection was defined as a recurrent disease episode with a different *M. tuberculosis* strain. Relapse was defined as a recurrent disease episode with the same *M. tuberculosis* strain found in the previous episode, assuming that reinfection with the same strain is not expected to be significant because strain diversity in this community is high [14]–[16]. The same definition was proposed by others [1],[4],[7],[9] New case was defined as a case without any previous treatment. Recurrence was considered any time after end of treatment of the previous episode. A period less than 3 months after the end of the first treatment and longer than 60 months was excluded for epidemiological statistics inferring shorten period could be failure of treatment in most of the cases, and a period longer than five years as a new case. World Health Organization definitions were used to determine treatment outcome. A patient was considered cured when initially smear-positive and who was smear-negative in the last month of treatment and on at least one previous occasion.

### Laboratory methods

#### Susceptibility test and DNA extraction

The isolates were cultured in Löwenstein Jensen solid medium. The drug susceptibility testing was performed according to the proportion method described by Canetti et al. [17]. Chromosomal DNA extraction was performed using the cetyltrimethylammonium bromide (CTAB) method as described by van Soolingen et al. [18].

#### Genotyping analysis

Spoligotyping was performed as described by Kamerbeek et al. with a commercial kit (Ocimum Biosolutions BV, India) and the results were compared to a public database available at: http://www.pasteur-guadeloupe.fr:8081/SITVIT_ONLINE[19],[20]. MIRU-VNTR was carried out by Genoscreen® using the commercial 24-loci format. If MIRU-VNTR has at least one locus of difference it was considered as different strains.

#### Statistical analysis

Statistical analysis was performed using SPSS software (SPSS Inc., Chicago, IL). The groups RC, RD and NC were compared by Chi-square, *t*-test. Logistic regression analysis was made in the cases where bivariate analysis showed significative differences. Statistical significance was considered if p <0.05 with 95% confidence intervals. To analyze possible confounders variables, multivariable analysis was performed.

## Results

**Figure 1:**
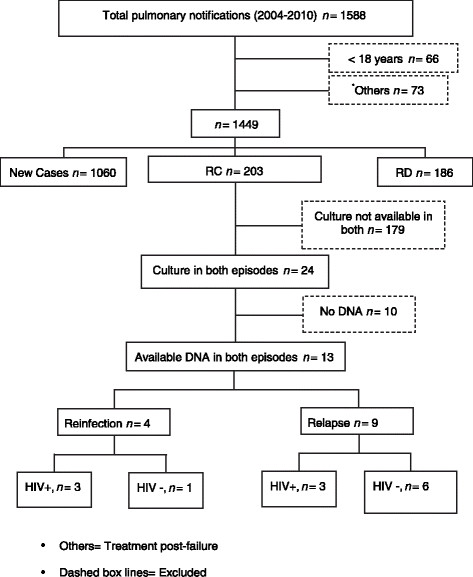
**The flow chart of tuberculosis patient selection.**

A total of 1522 adult patients started treatment for pulmonary TB between 2004 and 2010. Patients starting treatment after failure were excluded (73/4.6%). In the eligible group (1449), there were 1060 new cases (NC) (73.2%). Among the recurrent cases, 203 (14%) were RC and 186 (12.8%) were considered RD (Figure 1).

HIV positive status of the 3 groups NC, RC and RD were respectively 22.5, 37.7 and 43.3%. These groups were further compared about sex, age, smoking, alcohol abuse, drugs, and treatment compliance (Table 1).

**Table 1:** **Epidemiological and clinical characteristics of TB patients in the study period**

Total (*n* = 1449)		NC (%)	RC (%)	RD (%)
Total		n = 1060 (73.2)	n = 203 (14)	n = 186 (12.8)
	**Valid cases for NC/RC/RD**			
Sex Male	1060/203/186	67.2	63.1	75.3
HIV-positivity	1034/199/180	22.5^a^	37.7^a^	43.3
Alcohol abuse	965/171/152	35.5	35.7^b^	60.5^b^
Drug abuse	911/160/145	29.3	31.9	55.9
Smoke	971/172/153	63.4	65.7	73.9
Treatment noncompliance	940/170/163	28.5	34.7^c^	60.7^c^
Age years, median	1060/203/186	37 ± 13.9	39.6 ± 12.39	36.4 ± 10.7

NC = New Cases.

RC = Recurrence after cure.

RD = Recurrence after dropout.

^a^OR = 2.15 (1.55-2.97); p < 0.0001.

^b^OR = 2.60 (1.46-4.65); p = 0.001.

^c^OR = 2.15 (1.24-3.73); p = 0.006.

Comparing RC to NC, there were statistics differences in HIV status. These results indicate that HIV infection is an independent risk factor for recurrence of TB after cure [OR = 2.15 (1.55-2.97); p < 0.0001)]. Most of the 203 recurrences occurred later than 6 months after the end of the treatment of a previous episode 84.2% (n = 171/203). When considering only patients with a period between episodes less than 3 months (90 days) or higher than 60 months (1800 days); 121 of 203 recurrences; 97 (80.16%) occurred later than 6 months after the end of the treatment of a previous episode.

When RC and RD were compared, HIV status was not different in these groups. In the other hand, alcohol abuse and treatment noncompliance were statistically more frequent in those that dropped treatment [OR = 2.60 (1.46-4.65); p = 0.001)] and [OR = 2.15 (1.24-3.73); p = 0.006)] respectively.

Of the 203 recurrent TB cases, 24 had cultures done in the first and second episodes, but only 13 strains were available to culture for further DNA extraction. Spoligotyping and MIRU-VNTR were performed on available strains to differentiate whether the disease is due to reinfection or relapse. According to molecular techniques, in 4 (30.7%) patients the disease was attributed to reinfection and in 9 (69.3%), to relapse.

In the reinfection patients, according to MIRU-VNTR technique, there was an interval of 6 months or more between the first and second episode. In two of them, strains were isoniazid (INH) and rifampicin (RMP) resistant in both episodes. One patient had a INH resistant episode, followed by a susceptible strain 700 days later (patient 7 in Additional file 1: Table S1). Patient 11 had two susceptible strains in both episodes. Three patients were HIV positive and one negative (Additional file 1: Table S1).

In 9 (69.3%) cases the disease was attributed to relapse due to identical MIRU-VNTR profile. Among them, there were six patients that relapsed 6 months or more after the first episode. In this group, 6 cases were susceptible to all tested drugs, 2 cases were RMP and INH resistant and one was RMP resistant. Two patients were HIV positive and seven negative. No statistical differences between the two groups (relapse and reinfection) were found.

Among 26 spoligotyping performed, LAM and T family were the most common, 10 (38.5%). and 6 (23%) strains respectively.

## Discussion

In a previous study at the same city, recurrence of tuberculosis was estimated as 4.3% between 1989 and 1994 (0.55/100 person-years) [7]. Now, the high incidence of recurrence (26.8% of patients, n = 389) in the study period shows that TB is far from being controlled in this area and evidences the need to characterize this population. Since then, the incidence of HIV and TB had increased, achieving the second position in the unwanted ranking of brazilian capitals with one of the highest TB (104.6/100000 habitants) and TB/HIV (1/3 of them) incidence. Korenromp et al. found, in a study carried out in sub-Saharan Africa, rates of TB recurrence were up to 20/100 person years [21]. In a low incidence area, Nashville-Tennessee-USA, from 1431 reported TB cases, 20 (1.4%) cases recurred in the studied period [22]. It clearly shows the quality of TB control programs in these areas, as recurrence rates can be used to assess the effectiveness of TB control programs. In high TB incidence areas, recurrences are likely due to reinfection, related to the maintenance of infection source. However, in low incidence areas, when infection control is adequate, recurrences are likely due to relapse.

In thirteen publications from 1993 to 2001, reviewed by Lambert et al. [1], there was not a concordance about a standard in terms of timing to characterize recurrence. In two recently published reports, Millet et al. defined at least twelve months between two positive culture and for Luzze et al., it was considered any time after the end of the previous treatment [8],[9]. For epidemiological statistical proposal, to compare time between episodes it was considered from 3 months to five years, inferring shorten period could be failure of treatment in most of the cases, and a period longer than five years as a new case [7]. Nunn et al. reported 78% of 574 recurrences by relapses, occurred within 6 months of stopping treatment and 525 recurrences by reinfection (91%) within 12 months [23]. Recurrence due to relapse seems to occur closer to the time of cure [24]. In a large study made in a sub-Saharan TB-endemic country the median duration to TB recurrence was 9.9 months, in agreement with earlier African studies where 90% of clinical relapses occurred within 12 months after completing treatment [8],[25],[26]. In the same study time to reinfection was longer than to relapse (20.1 vs. 8.1 months p = 0.07) in HIV-positive patients [8]. In our study HIV positive patients had a longer time between episodes than HIV negative patients (p = 0.049) suggesting that reinfection may be more implicated than relapse in HIV positive and relapse may be more implicated in HIV negative. HIV positive patients attend more frequently the public health system, probably being more exposed to a new infection and are more susceptible to disease when infected. According Pettit et al. the higher rate of reinfection among HIV infected patients may be related to increase in exposure in high incidence areas and subsequent increased risk for disease progression [22]. The high number of recurrence in our study and the time between episodes suggest reinfection playing most of the cases as it is expected in a high burden area [1],[4],[7],[9], with a high strain diversity circulating [14]–[16].

Comparing RC to NC, HIV infection was independent risk factor for recurrence of TB after cure. Millet et al. reported more risk of TB recurrence in HIV positive patients [9]. Sonnenberg et al., Panjabi et al. and Pettit et al. report HIV infection as factor independently associated with recurrence [22],[27],[28]. Studies involving HIV positive patients have reported higher recurrence rates [9],[22],[29]. In our study 37.7% of recurrent patients after cure were HIV positive.

When RC and RD were compared, HIV status was not different in these groups. In the other hand, alcohol abuse and treatment noncompliance were statistic more frequent in those that dropped treatment than in RC group. In another study, the noncompliance was higher in alcohol abuse cases (20.9% vs. 9.9%; p = 0.01) [7]. Many studies were done to identify risk factors associated with TB treatment default. Alcohol use or alcohol abuse has been frequently reported as a risk factor for default [30]–[33].

As sputum culture in Brazil is not available for each patient starting treatment, only in few it was possible to obtain sputum in the first episode and in the recurrence of the disease for genotyping analysis [12]. Among the thirteen patients analyzed, in 4 (30.7%) patients the disease was attributed to reinfection and in 9 (69.3%), to relapse. There is no statistical difference among reinfection and relapse for epidemiological characteristics including HIV status.

The LAM family was the most frequent pattern involved in relapse, which is expected as this family was the most common in the study and it is very common in the study area as well as in Brazil [14]–[16]. The T family, the second most common family, is also, a very common profile causing TB disease in the South Brazil [14]–[16]. The study had no correlation among spoligopatterns families and resistance profile.

Despite no statistically difference among HIV status in the relapse group, probably due to the few number analyzed, there was a trend to an increase of relapse in HIV negative. Our findings are consistent with a study performed in an area of medium to high TB incidence, when HIV-negative patients are predominantly affected by relapse, while HIV positive patients are at risk of both relapse and reinfection [29],[34],[35]. The median time to TB relapse was 550 days. A very similar number reported was found by Pettit et al., when relapse was 412 days, despite this study was performed in low TB incidence area, approximately 5/100 000 against Porto Alegre area which TB incidence is approximately 160/100 000 [22].

## Conclusions

Considering the high incidence of recurrence, the period longer than 6 months after the previous episode suggests reinfection as a major cause for recurrence in a high setting of TB and HIV. HIV is the sole more important associated factor for recurrence after cure and alcohol abuse for treatment dropout. These finding points to the need for another approach to manage TB in order to decrease the chance for exposure especially in vulnerable people with increased risk of developing disease and to improve DOTS strategy to deal with factors associated to treatment noncompliance including psychological and social worker assistance.

## Authors’ contributions

GU conceived the study, analyzed the data, carried out statistical analysis and drafted the manuscript. AWR carried out experimental procedures, analyzed the data and revised the manuscript. LSE carried out experimental procedures. FSS carried out experimental procedures and revised the manuscript. PDP carried out statistical analysis, analyzed the data and revised the manuscript. ERDC analyzed the data and drafted the manuscript. MLRR conceived the study, revised experimental procedures and revised the manuscript. All authors read and approved the final manuscript.

## Additional file

## Electronic supplementary material

Additional file 1: Table S1.: Genetic characteristics of strains infecting TB recurrent patients in the study period. (DOC 102 KB)

Below are the links to the authors’ original submitted files for images.

Authors’ original file for figure 1
