# Supplementary material for: Tuberculosis recurrence in a high incidence setting for HIV and tuberculosis in Brazil
Source: BMC Infect Dis. 2014 Oct 24;14:548. doi: 10.1186/s12879-014-0548-6 (PMC4215011; doi:10.1186/s12879-014-0548-6)
Supplement: Supplementary file 1 — Additional file 1: Table S1.: Genetic characteristics of strains infecting TB recurrent patients in the study period. (DOC 102 KB) [file 12879_2014_548_MOESM1_ESM.doc]

**Additional file 1: Table S1**

| **P^1^** | **I** | **E** | **R** | **S** | **Days*** | Spoligopattern |  | **ST** | **Family** | 24 MIRU-VNTR type | | | | | | | | | | | | | | | | | | | | | | | |  |
| --- | --- | --- | --- | --- | --- | --- | --- | --- | --- | --- | --- | --- | --- | --- | --- | --- | --- | --- | --- | --- | --- | --- | --- | --- | --- | --- | --- | --- | --- | --- | --- | --- | --- | --- |
| 1 | S | S | S | S | 300 |  | S | 17 | LAM2 | 2 | 5 | 3 | 1 | 4 | 2 | 2 | 3 | 3 | 3 | 5 | 2 | 2 | 5 | 1 | 2 | 2 | 1 | 2 | 3 | 4 | 2 | 2 | 3 | S |
|  | S | S | S | S |  |  |  | 17 | LAM2 | 2 | 5 | 3 | 1 | 4 | 2 | 2 | 3 | 3 | 3 | 5 | 2 | 2 | 5 | 1 | 2 | 2 | 1 | 2 | 3 | 4 | 2 | 2 | 3 |  |
| 2 | S | S | S | S | 180 |  | S | 17 | LAM2 | 2 | 5 | 3 | 1 | 4 | 2 | 2 | 3 | 3 | 3 | 5 | 2 | 2 | 5 | 1 | 2 | 2 | 1 | 2 | 3 | 4 | 2 | 2 | 3 | S |
|  | S | S | S | S |  |  |  | 17 | LAM2 | 2 | 5 | 3 | 1 | 4 | 2 | 2 | 3 | 3 | 3 | 5 | 2 | 2 | 5 | 1 | 2 | 2 | 1 | 2 | 3 | 4 | 2 | 2 | 3 |  |
| **3+** | S | S | S | S | 480 |  | S | 65 | T1** | 2 | 4 | 4 | 3 | 4 | 5 | 3 | 3 | 2 | 3 | 7 | 3 | 2 | 5 | 4 | 2 | 2 | 1 | 3 | 3 | 4 | 2 | 3 | 3 | S |
|  | S | S | S | S |  |  |  | 65 | T1 ** | 2 | 4 | 4 | 3 | 4 | 5 | 3 | 3 | 2 | 3 | 7 | 3 | 2 | 5 | 4 | 2 | 2 | 1 | 3 | 3 | 4 | 2 | 3 | 3 |  |
| 4 | R | S | R | S | 140 |  | S | 104 | U | 2 | 3 | 2 | 3 | 4 | 4 | 3 | 3 | 4 | 4 | 7 | 2 | 2 | 6 | 2 | 2 | 2 | 1 | 2 | 3 | 4 | 2 | 1 | 1 | S |
|  | **R** | S | **R** | S |  |  |  | 104 | U | 2 | 3 | 2 | 3 | 4 | 4 | 3 | 3 | 4 | 4 | 7 | 2 | 2 | 6 | 2 | 2 | 2 | 1 | 2 | 3 | 4 | 2 | 1 | 1 |  |
| **5+** | **R** | S | **R** | S | 180 |  | S | 93 | LAM5 | 2 | 5 | 3 | 1 | 4 | 4 | 1 | 3 | 4 | 4 | 8 | 2 | 2 | 6 | 1 | 2 | 2 | 1 | 2 | 2 | 4 | 1 | 2 | 4 | D |
|  | **R** | S | **R** | S |  |  |  | 93 | LAM5 | 2 | 5 | 3 | 1 | 4 | 4 | 1 | 3 | 4 | **3+4** | 8 | 2 | 2 | 6 | 1 | 2 | 2 | 1 | 2 | 2 | 4 | 1 | 2 | 4 |  |
| 6 | S | S | S | S | 60 |  | S | 65 | T1** | 2 | 4 | 4 | 3 | 4 | 5 | 3 | 3 | 2 | 3 | 7 | 3 | 2 | 5 | 4 | 2 | 2 | 1 | 3 | 3 | 4 | 2 | 3 | 3 | S |
|  | S | S | S | S |  |  |  | 65 | T1** | 2 | 4 | 4 | 3 | 4 | 5 | 3 | 3 | 2 | 3 | 7 | 3 | 2 | 5 | 4 | 2 | 2 | 1 | 3 | 3 | 4 | 2 | 3 | 3 |  |
| **7+** | R | S | S | S | 700 |  | D | 42 | LAM9 | 2 | **5** | **3** | **1** | 4 | 5 | **2** | 3 | **5** | **4** | **8** | **2** | 2 | **6** | **1** | 2 | 2 | 1 | **2** | 3 | 4 | **1** | **2** | **5** | D |
|  | S | S | S | S |  |  |  | 65 | T1** | 2 | **4** | **4** | **3** | 4 | 5 | **3** | 3 | **2** | **3** | **7** | **3** | 2 | **5** | **4** | 2 | 2 | 1 | **3** | 3 | 4 | **2** | **3** | **3** |  |
| 8 | S | S | S | S | 300 |  | S | NI |  | 2 | 5 | 2 | 4 | 3 | 3 | 3 | 3 | 2 | 4 | 5 | 3 | 2 | 6 | 2 | 2 | 2 | 1 | 2 | 3 | 4 | 2 | 3 | 3 | S |
|  | S | S | S | S |  |  |  | NI |  | 2 | 5 | 2 | 4 | 3 | 3 | 3 | 3 | 2 | 4 | 5 | 3 | 2 | 6 | 2 | 2 | 2 | 1 | 2 | 3 | 4 | 2 | 3 | 3 |  |
| 9 | R | S | S | S | 3300 |  | S | 65 | T1** | 2 | 4 | 4 | 3 | 4 | 5 | 3 | 3 | 2 | 3 | 7 | 3 | 2 | 5 | 4 | 2 | 2 | 1 | 3 | 3 | 4 | 2 | 3 | 3 | S |
|  | **R** | S | S | S |  |  |  | 65 | T1** | 2 | 4 | 4 | 3 | 4 | 5 | 3 | 3 | 2 | 3 | 7 | 3 | 2 | 5 | 4 | 2 | 2 | 1 | 3 | 3 | 4 | 2 | 3 | 3 |  |
| **10+** | **R** | S | R | S | 90 |  | S | 60 | LAM4 | 1 | 3 | 3 | 1 | 3 | 4 | 1 | 2 | 5 | 4 | 8 | 2 | 2 | 4 | 1 | 2 | 2 | 1 | 2 | 2 | 4 | 1 | 2 | 5 | S |
|  | **R** | S | **R** | S |  |  |  | 60 | LAM4 | 1 | 3 | 3 | 1 | 3 | 4 | 1 | 2 | 5 | 4 | 8 | 2 | 2 | 4 | 1 | 2 | 2 | 1 | 2 | 2 | 4 | 1 | 2 | 5 |  |
| **11+** | **R** | S | **R** | S | 180 |  | D | NI |  | 2 | 5 | **3** | 3 | **4** | **4** | 3 | 3 | **3** | **2** | **4** | **2** | 2 | **6** | **2** | 2 | **2** | 1 | 2 | **2** | **4** | 2 | **1** | **1** | D |
|  | **R** | S | **R** | S |  |  |  | NI |  | 2 | 5 | **2** | 3 | **5** | **5** | 3 | 3 | **2** | **3** | **7** | **3** | 2 | **3** | **4** | 2 | **1** | 1 | 2 | **3** | **2** | 2 | **3** | **3** |  |
| 12 | S | S | S | S | 270 |  | S | 58 | T5**** | 2 | 5 | 2 | 5 | 3 | 3 | 3 | 3 | 2 | 4 | 5 | 3 | 2 | 6 | 2 | 2 | 2 | 1 | 2 | 3 | 4 | 2 | 2 | 4 | S |
|  | S | S | S | S |  |  |  | 58 | T5**** | 2 | 5 | 2 | 5 | 3 | 3 | 3 | 3 | 2 | 4 | 5 | 3 | 2 | 6 | 2 | 2 | 2 | 1 | 2 | 3 | 4 | 2 | 2 | 4 |  |
| 13 | S | S | S | S | 1825 |  | D | 137 | X2 | 2 | 5 | **4** | **3** | 4 | **3** | 3 | **3** | **2** | 4 | **8** | **3** | 2 | **5** | **4** | 2 | **1** | 1 | **1** | 3 | **6** | 2 | **4** | 3 | D |
|  | S | S | S | S |  |  |  | NI | LAM | 2 | 5 | **2** | **6** | 4 | **2** | 3 | **1** | **3** | 4 | **7** | **1** | 2 | **6** | **1** | 2 | **2** | 1 | **2** | 3 | **4** | 2 | **2** | 3 |  |

P^1^= Patient (**+** = HIV status)

I= Isoniazid; E= Etambutol; R= Rifampicin; S= Streptomicin

S= Susceptible; R= Resistant

D= Different; S= Same

NI= Not Identified by SITVITWEB

* Days between episodes.

** T1 (T4-CE1 ancestor?)

*** U (LAM3?)

**** T5_MAD2
