# Supplementary figures and images for: Tuberculosis recurrence in a high incidence setting for HIV and tuberculosis in Brazil
Source: BMC Infect Dis. 2014 Oct 24;14:548. doi: 10.1186/s12879-014-0548-6 (PMC4215011; doi:10.1186/s12879-014-0548-6)

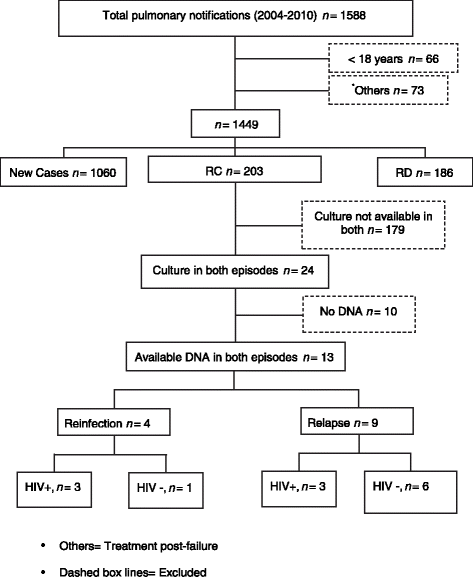

Supplement: Supplementary file 2 — Authors’ original file for figure 1 [file 12879_2014_548_MOESM2_ESM.gif]
